# On the Importance of the Early Recognition and the Repression of Mental Disease in Its Incipient Stages

**Published:** 1881-06

**Authors:** Edward C. Mann

**Affiliations:** 28 West 30th St., New York City. Physician in Chief to Sunnyside—A private hospital for diseases of the Nervous System, Dipsomania and Opium Habit


### Independent Practitioner.

Vol. II.——*June*, 1881.——No. 6.

ARTICLE I.

### ON THE IMPORTANCE OF THE EARLY RECOGNITION AND THE REPRESSION OF MENTAL DISEASE IN ITS INCIPIENT STAGES.

BY EDWARD C. MANN, M. D., 28 West 30th St., New York City.
Physician in Chief to Sunnyside—A private hospital for diseases of the Nervous System, Dipsomania and Opium Habit.

There exists in insanity, in common with other cerebral diseases, a stage of incubation, in which the insanity is not yet characterized, and in which it commences with incomplete manifestations. It is an equivocal state, differing but little from perfect sanity, but is the earliest phase of mental alienation, and if recognized by the general practitioner and properly treated in this incipient stage, subsequent trouble might be averted. As a primary proposition for the consideration of the general practitioner, who must, for the prevention of insanity, understand the principles which are the foundation of Psychological medicine, we would say, that in his relations with the young in the educational period, he should remember that precocity is a sign of biological inferiority, and that the precocity of organisms and organs is in an inverse ratio to the extent of their evolution.

The Psycho-neuroses which attack an intact brain, often commence in intellectual exertion of the exhausted brain, the exhaustion being induced by taking up too great a variety of subjects for study, during the educational period of life. We have, as a result, a passive dilatation of the blood vessels of the brain, connected with disturbances of nutrition, or an anæmia of the brain may produce grave nutritional disturbances in the ganglion cells of the certex of the brain. We get as a result of either of these states habitual headache, and a loss of intellectual tone. Perhaps these slight disturbances may not attract particular attention, or such cases may be dismissed with some simple prescription, but let it be remembered that one of the gravest and most incurable of nervous disorders, progressive paralysis, commences in just this insidious manner, as a vaso-motor disturbance of nutrition of the cortical portion of the brain, where the vessels of the pia-mater soon get into a state of passive dilatation and the disease thus established proceeds to its termination. Great attention should, therefore, be paid to the very earliest indication of brain exhaustion, whether in school children and in the young during the whole educational period of life, or in those of more advanced age, where the earliest symptoms are those of nervous exhaustion, which, if not checked, rapidly lapses into actual mental disorder. The brain may *not* be intact, but may be predisposed to the acquisition of mental disease by hereditary, or acquired vices of conformation or nutrition, and then constitutional affection, insanity, moral insanity, the monomanias, epileptic insanity, hysterical insanity, hypochondriacal insanity and periodical insanity may appear, if nervous exhaustion should appear and run an unchecked course, or if the early symptoms of any of these states be disregarded as matters of slight importance.

If a patient complains of general malaise, impaired nutrition and assimilation, if we find muscular atonicity changing the facial expression, if neuralgia is present, if we find cerebral anæmia, if our patient manifests mental depression, and, above all, if he is sleepless, we have induced a rapid state of prostration, which will soon precipitate our patient into active insanity, if these symptoms are not most effectively combated. Irritability and distrust are grave psychical symptoms in asthenic cases.

If we have cerebral hyperæmia in our patient, headache may then be a prominent symptom. We must recognize these symptoms as those of a grave nervous prostration which, unchecked, lapses into actual insanity with great readiness. I never like to see neuralgia developing in such cases, as it is, when not malarial, very often a premonitory symptom of impending mental disturbances when associated with other symptoms of nervous prostration. Profuse perspirations also are found in connection with nervous prostration, and occur at any hour of the day or night. A loss of the normal elasticity of the skin is another prominent symptom of disordered nervous action. Arsenic as a remedy in this latter class of cases is very valuable.

In children, or young people from fifteen to twenty years, very grave psychical disorders may appear which require the promptest treatment. The history of such patients will usually be, that during childhood they have been excessively nervous, and have perhaps had convulsions in infancy. They have been very emotional children, suffering from night terrors. There are periods of marked mental inactivity, alternating with a hyper-activity of the mental functions, and such patients do not take or manifest a normal or healthy interest in their surroundings. If hysterical girls, they may neither eat or sleep for some days at a time. There are no suicidal or homicidal tendencies in these cases, but a disposition to recurrent mania. In the menstrual psychoses of young girls and women, the psychic disorders which come on at these times, are, I think, more than a natural exaggeration of the nervous excitability, which we may naturally expect in a female at this period. It is a true periodic insanity in many cases, an acute psychosis, with the intellectual centres involved. They are vaso-motor neuroses with recurrent cerebral hyperæmia. We find this form of periodical insanity at any epoch of sexual life, and there is marked physical and mental prostration in the intervals between the paroxysms, and we should combat these states by every means in our power. I remove uterine trouble, if any exist, use the constant current of electricity to the central nervous system to improve its nutrition, and use sodium bromide and ergot in combination. The mono-bromide of camphor in Clin's Capsules of four grains each, is also very valuable in some of these cases. I also, for one week preceding the appearance of the menses in such women, employ cerebral electrization daily, using the constant current, which possesses the power of combating and perfectly antagonizing the various congestive states, which, unchecked, lead to insanity. Of all the cases in which I am accustomed to use electrization of the brain, none give more gratifying results than these periodic menstrual psychoses in women. A marked tendency to sleep, even in cases which have been sleepless for days, follows these applications. I have never seen any evil results from the use of moderately strong currents judiciously applied, and, on the contrary, I have more than once prevented the access of insanity by this means. Certainly, I know, that in certain cases where there had been a recurrent, periodical menstrual psychosis or mania, this treatment has resulted in my hands in the complete cure of the patient, when conjoined with the proper medicinal treatment, so that I most earnestly, and from experience, advocate its use. In a paper in the *New York Medical Gazette*, I have given my views at length on this subject, of the value of the constant current of electricity as an application to antagonize cerebral hyperæmia and ward off impending mental disease.

I would insist upon the point, that in young ladies especially, their mental future depends very largely upon the nervous and physical strength which they attain before the age of twenty-one. Many patients are brought to me suffering from nervous prostration and protracted headache near the monthly menstrual epoch, all on account of too great intellectual exertion, inducing a very nervous and hysterical condition. We too often sacrifice the *constitution* to what we deem *educational necessities. I deem the necessity in a young girl*, to have plenty of bone, blood and muscle, and to be well developed, with an accurate balance between the physique and the nervous system, and if something has to be sacrificed, let it be some of her education and *not* some of her mental and physical health.

Insanity will just as surely follow neglect of mental hygiene as the zymotic diseases follow neglect of sanitary precautions, and we too often overlook this fact, for the reason that the incubating stage of insanity may be, and often *is*, long and insidious, and easily overlooked by one who is not a student of psychological medicine. It is very easy to ruin the delicate tissue of the brain by over-straining it when exhausted. There are too many young brains not strong and vigorous, but unstable, and subject to irregular and uncertain action, which have been rendered so by an entirely false system of education.

There is a great deal of brain fatigue among professional and business men, resulting from the preponderance of waste and repair which induces grave nervous prostration.

Such patients complain of a loss of physical and mental power, and of an inability to do what they could do when well, and these same patients exhibit exaggerated sensibility, being very easily affected by trivial impressions. Such patients suffer much from vertigo and confusion of mind, owing to an impaired nutrition of the brain and spinal cord, and a diminution of vascular tonus.

One very important set of symptoms to early recognize and combat, are those characteristic of cerebral syphilis. In these cases we have a deep-seated headache, of extraordinary intensity, with *nocturnal exacerbation*, and of long duration. The headache is the premonitory symptom of very grave cerebral mischief, which we may ward off if we recognize its significance.

As the results of the cerebral congestion of specific origin we have vertigo and dullness, temporary disorders of the special senses and momentary impairments of the intellect. These symptoms, at first transitory, may become permanent by inattention. Congestive attacks of greater intensity, even attaining the grade of apoplectic fits, may now occur. In the gravest forms of specific cerebral disease, an apoplectiform attack, followed by fatal coma, may usher in the attack with no premonitory symptoms. Epilepsy, if commencing after twenty years of age, is due, probably, to specific brain disease, and is often preceded by the premonitory headache, of which I have spoken. In these cases I always put a patient immediately on energetic anti-syphilitic treatment, as I care little about the history. The epilepsy is, to me, evidence of the existence of the disease. The mental symptoms, when insanity appears, are those of exaltation—delirium and mania. The gravest forms of this disorder yield rapidly to appropriate treatment. If we find in a patient, a male more particularly, persistent mental dullness and muscular feebleness, existing as vague, undefined symptoms, it is always well to examine that patient's history pretty thoroughly, and a specific course of treatment may very likely prevent in such a patient the invasion of insanity. We must not promise perfect recoveries in these cases of cerebral syphilis, for some never recover, and there may be incomplete recoveries. In a certain proportion of cases, however, we may get a rapid and brilliant cure. Cold douches are very valuable in cerebral syphilis, as an adjunct to specific treatment and should never be omitted.

I am more inclined to think that syphilitic brain disease is overlooked, than that it is so very rare as some authors claim.

I have detailed the symptoms of the gradual breaking down of the nervous system, causing nervous prostration, and incipient insanity, and would now briefly state my treatment of such cases. We must secure for our patient good, refreshing sleep, and take him away for a time from business cares and anxieties, and, if a woman, give her *rest.* If the condition is asthenic, alcoholic stimulants are indicated, to ward of the cerebral anæmia, which, if not relieved, will bring on an attack of mania. Strychnine is also indicated in these anæmic states. I usually use the citrate of iron, quinine and strychnia, rest, massage and electricity, together with a milk punch three times a day. If there is a condition of cerebral congestion, I employ, as I have said, the constant current of electricity to the brain to antagonize the congestive states. The bromide of zinc, commencing with ¼ to 1 gr. doses. and the hydro-bromate of quinine, are both useful in cerebral congestion, but we are also apt to have an anæmic and asthenic state of the system, especially in women. In these conditions, quinine is one of the best nerve tonics, and may be given in 1 or 2 gr. doses before each meal. Arsenic is also very valuable indeed. By appropriate and judicious treatment we may get a perfect cure in the incipient stages of insanity, and generally with no fear of a relapse.

Modern nervous diseases are rapidly increasing and multiplying, and our morbid nervousness, which is developing itself in modern society, is making itself manifest by a great increase in neuralgia, sick headache, dyspepsia and nervous exhausion. The increasing nervousness of this country is most clearly evinced by the connection with and influence of the nervous system on other diseases not properly nervous. Thus, in diabetes, the nervous system is in intimate relation with the disease, and I consider that it is often induced by mental anxiety and distress or by sudden fear and shock. It would appear to be advancing *pari passu* with the increase of nervous diseases. It is a disease decidedly more common than it used to be, and there can be no doubt but that its greater prevalence is due to our present state of civilization. Bright's disease of the kidneys, nephritis, and granular kidney are also caused and aggravated by mental worry and anxiety. Heart diseases are also increasing steadily, particularly those of neurotic origin and nature. The neurotic circle in society is increasing out of proportion to the increase of populaton, as well as the distinctly insane circle of society. The causes of all this nervousness are due, first, to the increasing complexity of the nervous system, and, secondly, the increased complexity of life. There is an elaboration in brain structure, and in the way of a finer architecture of our brains, new phases of intelligence and new proclivities to nervous disease. Our brains are finer in structure and more subtle in mechanism, but instability is the result. The conditions of modern life, which act on our complex and excitable nervous systems, cause our increased nervous disease, and even mental disease itself. As I have said in the beginning of this paper, modern systems of education are also influential in promoting nervousness, and in contributing to the increase of mental and nervous diseases, and there are serious dangers lurking in cur present teaching processes. In many of my patients I have traced sleeplessness, night terrors, somnambulism, epilepsy, hydrocephalus, and other troubles to educational pressure unwisely applied to delicate children. It is a point of importance to understand that brain tissue degenerations and mental diseases may be separated by long intervals of time, from the too premature and intense stimulation of the brain which cause these nervous diseases. Hydrocephalus, however, is a nervous disease which shows itself at once from over-stimulation of the brain in the young, and of late years the increase in deaths from this disease has not been among infants, but among children and young people, from five to twenty years, in the educational period of life. This is a very significant fact. More remotely, as a cause of over-stimulating the brain by education, we meet with the preponderance of nervous diseases in the refined and cultivated classes.

The functional activity of the brain is established at different epochs and perfected at different rates. By cautious stimulation of the brain we bring it to its highest development. By undue haste we ruin its functional activity forever, and never have a sound and vigorous brain. The whole future complexion of mental life is in a great part determined by the impression made on the sensory centres of the brain when they are undergoing development. We must aim, therefore, in our system of education, at a harmonious development of body, brain and mind alike, and we shall then attain progress, with health combined.

